# Accuracy of Portable Face-Scanning Devices for Obtaining Three-Dimensional Face Models: A Systematic Review and Meta-Analysis

**DOI:** 10.3390/ijerph18010094

**Published:** 2020-12-25

**Authors:** Hang-Nga Mai, Jaeil Kim, Youn-Hee Choi, Du-Hyeong Lee

**Affiliations:** 1 Institute for Translational Research in Dentistry, Kyungpook National University, Daegu 41940, Korea; mai.hang.nga1403@gmail.com (H.-N.M.); cyh1001@knu.ac.kr (Y.-H.C.); 2 School of Computer Science and Engineering, Kyungpook National University, Daegu 41940, Korea; jaeilkim@knu.ac.kr; 3 Department of Preventive Dentistry, School of Dentistry, Kyungpook National University, Daegu 41940, Korea; 4 Department of Prosthodontics, School of Dentistry, Kyungpook National University, Daegu 41940, Korea

**Keywords:** accuracy, three-dimensional face model, portable facial scanning, systematic review, meta-analysis

## Abstract

The use of three-dimensional face-scanning systems to obtain facial models is of increasing interest, however, systematic assessments of the reliability of portable face-scan devices have not been widely conducted. Therefore, a systematic review and meta-analysis were performed considering the accuracy of facial models obtained by portable face-scanners in comparison with that of those obtained by stationary face-scanning systems. A systematic literature search was conducted in electronic databases following the Preferred Reporting Items for Systematic Reviews and Meta-Analyses guidelines for articles published from 1 January 2009 to 18 March 2020. A total of 2806 articles were identified, with 21 articles available for the narrative review and nine studies available for meta-analysis. The meta-analysis revealed that the accuracy of the digital face models generated by the portable scanners was not significantly different from that of the stationary face-scanning systems (standard mean difference (95% confidence interval) = −0.325 mm (−1.186 to 0.536); z = −0.74; *p* = 0.459). Within the comparison of the portable systems, no statistically significant difference was found concerning the accuracy of the facial models among scanning methods (*p* = 0.063). Overall, portable face-scan devices can be considered reliable for obtaining facial models. However, caution is needed when applying face-scanners with respect to scanning device settings, control of involuntary facial movements, landmark and facial region identifications, and scanning protocols.

## 1. Introduction

The assessment of the facial structure is important in the dental and medical fields of craniomaxillofacial surgery, orthodontics, prosthodontics, orthopedics, and forensic medicine [1,2,3]. Accurate acquisition of face anatomical information significantly contributes to enhancing the reliability of treatment planning, the predictability of treatments, and the quality of results analyses [2,4,5]. Attempts have been made to quantitatively and qualitatively assess the soft-tissue profile of the human face to support a description of facial size and shape [6,7]. The most classical method employed is facial anthropometry, by which clinical indices are measured with the use of a caliper, steel tapes, and protractors [8]. Although facial anthropometry is a direct and simple method, its reliance on manual tasks leads to human errors and its success depends on the operator’s proficiency. Moreover, the time used for conducting measurements in the clinic may lead to discomfort among patients, especially when multiple indices are evaluated.

The use of a two-dimensional (2D) digital photograph is a basic approach to bring infographics into the realm of face assessment [1,4,9]. The desired measurements are visualized in 2D photos that are used to communicate between clinicians and patients. However, the human face is a complex geometric structure with different depths and textures, thus, it is difficult to realistically simulate the face in a 2D image. In particular, this method could be prone to error in the assessment of a facial deformity or face asymmetry, because 2D analyses are not appropriate for evaluating the volume of facial portions that are related to neuromuscular balance and facial harmony [10]. As a consequence, the prediction of results and the prognosis of treatments can be limited in this method.

To overcome the shortcomings of facial anthropometry and 2D face assessment techniques, three-dimensional (3D) optical scanning methods, such as stereophotogrammetry [11,12,13,14], laser scanning [1,15,16], and structure light scanning, were introduced [17,18]. These optical scanning methods provide 3D replication of the facial structure surface by generating a digital face model that can be coupled with a radiographic image of underlying bones and analytic software for 3D face analysis and virtual treatment planning or realistic surgery simulation [10,19,20,21]. Furthermore, the collected scan data can be utilized for multidisciplinary purposes in research and education as well as treatment.

The accuracy of the 3D optical scanning systems is fundamental and is of interest [22,23]. Based on the type of equipment of the optical scan devices, the scanning systems can be divided into two classifications, namely, stationary systems, where the optical devices are fixed on tripods or adjustable frames, and portable/hand-held systems, where the scanners are movable in real time around the target objects [24]. Stationary systems are widely used in diverse fields associated with facial anatomy, changes in facial shape with growth, orthopedic and plastic surgery, and orthodontic and prosthodontic treatments [25,26]. Nonetheless, the stationary systems have drawbacks derived from both their high encumbrance and their operating methods that require frequent calibration [7,24,27]. These limitations accelerated the development of portable systems and, gradually, portable systems encompassing wireless and compact optical devices were increasingly adopted [12,25].

Although the accuracy of stationary face-scanning systems was reported to be clinically acceptable, the reliability of portable systems has not been systematically clarified [18,22,23,28,29]. This article therefore aimed to review the accuracy of 3D human face models generated from portable 3D face-scanning systems in comparison with those created by stationary systems and to summarize the current knowledge about the various portable systems.

## 2. Materials and Methods

### 2.1. Search Strategy

**Figure 1:**
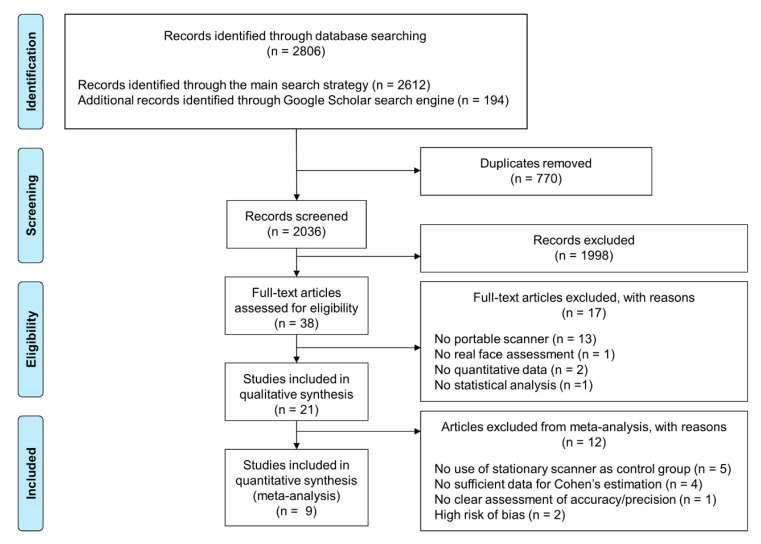
PRISMA flow diagram summarizing the literature search and selection process.

This study followed the guidelines provided by the Preferred Reporting Items for Systematic Reviews and Meta-analyses (PRISMA) [30]. The study was designed considering the population, intervention, comparison, and outcomes (PICO) question method, asking whether digital models of the human faces (P) created by portable 3D face scanners (I) comparable to those created by stationary 3D face-scanning systems (C) in terms of accuracy (O). The main search strategy was assembled considering a combination of qualified Medical Subject Headings (MeSH) terms and deployed in PubMed (Medline). Alongside this, individual search strategies were formulated and applied in several other electronic databases, including Scopus, Science Direct, and the Cochrane Library. The Google Scholar search engine was used to find additional articles by combining the free-text search terms with the main search strategy. The formulated Boolean operators incorporated in each searching strategy are presented in Supplementary Materials Table S1. The PRISMA flow diagram that illustrates the search and evaluation process is depicted in Figure 1.

### 2.2. Inclusion and Exclusion Criteria

The present review consisted of original studies that evaluated the dimensional accuracy of the 3D facial models obtained from digital face-scanning systems. Accuracy was determined by the presence or absence of a discrepancy between the facial virtual model generated by a digital facial scanning system and a reference/standard model. The deviations were evaluated by inter-landmark linear distances and/or global surface-to-surface deviation. The inclusion and exclusion criteria for study selection are described in Table 1. All relevant English articles published from 1 January 2009 to 18 March 2020 in peer-reviewed journals were considered for inclusion.

### 2.3. Study Selection and Data Extraction

Two independent reviewers (Hang-Nga Mai and Du-Hyeong Lee) participated in screening and selecting the studies in accordance with the inclusion and exclusion criteria. The first step of the screening process was to identify relevant articles based on the information provided in their titles and abstracts. Thereafter, the full-text versions of articles agreed on by both reviewers as relevant were obtained and screened again to evaluate further adherence to the inclusion criteria. A calibration exercise involving the two reviewers was conducted and screening was started when their agreement was more than 90%. Disagreements between the two reviewers were resolved by discussion. The inter-rater variability was assessed by the Cohen’s kappa coefficient. Subsequently, the following data were extracted from the eligible studies: author(s), year of publication, study purpose, sample features, scanning methods, reference standard for validation, types of measurement performed, number of measurements, measurement results, and conclusions.

### 2.4. Quality Assessment

A risk-of-bias assessment was performed using the Quality Assessment Tool for Diagnostic Accuracy Studies-2 (QUADAS-2) [31]. This tool includes questions related to four bias domains, including patient selection, index test, reference standard, and flow and timing. When one or more of the key domains were scored as high risk, the study in question was judged as showing a high risk of bias in its overall judgment. When more than two key domains were rated as unclear, the study was regarded as having an unclear risk of bias. Each study was independently graded. The weighted bar-plots of the distribution of risk-of-bias judgments and traffic-light plots of the specific domain-level judgments for each study were created within each bias domain using the ROBVIS package for R (R version 3.6.0 software program; R Foundation for Statistical Computing, Vienna, Austria) [32].

### 2.5. Data Analysis

To evaluate the accuracy of the scanners, the standard mean difference (SMD) in each eligible study was calculated by the following equation:

(1) $$\mathrm{SMD}=\frac{\mathrm{Difference}\;\mathrm{in}\;\mathrm{mean}\;\mathrm{values}\;\mathrm{between}\;\mathrm{groups}}{\mathrm{Standard}\;\mathrm{deviation}\;\mathrm{of}\;\mathrm{measurements}}$$

A value of 0 for the SMD indicated that the effects of the digital face model generated from the 3D face-scanning system and the reference image were the same.

To analyze the effect size, a fixed-effects model or a random-effects model was selected based on the heterogeneity among studies, while the inverse variance-weighted estimation method was adopted for weighted estimation. The heterogeneity test was evaluated by the Higgins I^2^ statistic [33]:

(2) $$I^{2}=100\%\times \left(Q-df\right)/Q$$

where *Q* is the chi-squared statistic and *df* is the degree of freedom of the *Q* statistic.

An I^2^ value less than 25% was considered to show weak heterogeneity, an I^2^ value of 50% was average, and a value greater than 75% indicated strong heterogeneity. When the data were considered statistically heterogeneous, a random-effects model was selected. In subgroup analyses, the data were divided based on the type of portable face-scanning system involved. All analyses were performed using the Meta package for R and the significance level was set at 0.05. The pooled estimates for the global group and subgroups, which are the outcomes of the meta-analysis, were presented using forest plots.

### 2.6. Publication Bias

Publication bias was firstly detected and visually inspected using funnel plots. Second, Egger’s test was used to test for publication bias statistically. When publication bias was detected, the trim-and-fill method was used to correct the bias.

## 3. Results

### 3.1. Search Results

The database search initially identified a total of 2806 relevant articles. After removing 770 duplicated articles, 2036 articles remained underwent a review of their titles and abstracts. After the exclusion of 1998 irrelevant articles, 38 studies were assessed for further eligibility. Full-text reading led to the exclusion of 17 articles according to the inclusion and exclusion criteria, leaving 21 articles available for the narrative review and nine studies available for meta-analysis. The inter-rater agreement for the screening process was 96.43% (*k* = 0.90). The search results are described in the PRISMA flowchart (Figure 1).

### 3.2. Quality Assessment and Applicability Concern

**Figure 2:**
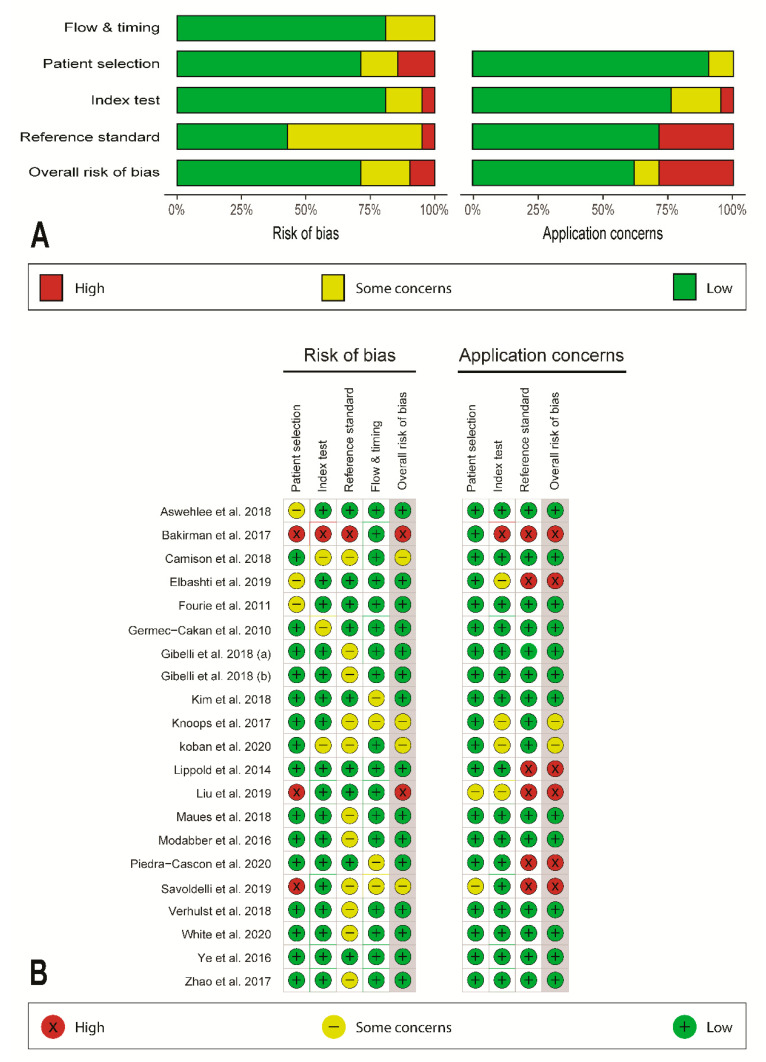
Risk of bias and application concerns according to the QUADAS-2 guidelines. (**A**) Weighted bar-chart; (**B**) traffic light plots.

Figure 2 showed the results of the quality assessment by QUADAS-2. Among 21 articles, 15 papers had a low risk of bias [1,6,11,13,18,25,29,34,35,36,37,38,39,40,41], four articles displayed some level of unclear risk of bias [12,17,28,42], and two articles had a high risk of bias [15,43]. Considering applicability concerns, 13 articles exhibited a low level of concern [1,6,11,12,13,25,34,35,36,38,39,40,41], two articles showed an unclear level of concern [17,28], and six articles showed a high level of concern [15,18,29,37,42,43]. Major bias was found in the patient selection domain because some studies were not clear in method to provide random samples or used a small number of included volunteers/patients [11,15,18,34,41,42,43]. As for the index test domain, most studies provided adequate manufacturer information and parameter setups for the tested scanning systems. One study [15] did not explicitly provide the manufacturer information but did encode the tested systems as systems A, B, and C and provided the technical properties of the systems. Finally, concerning the reference standard domain, most studies used direct anthropometry or industrial, high-resolution stereophotogrammetry to generate the reference models.

### 3.3. Characteristics of Included Studies

The characteristics of the included studies are summarized in Table 2. Among 21 studies, 18 studies were conducted using adult volunteers with a mean age of 31.54 ± 7.91 years (range: 21–62 years). Meanwhile, the number of participants ranged from 2 to 50 people among the studies. Impressions of patient faces were assessed in two studies [11,18] and human cadaver heads were assessed in one study [34]. Five studies [12,13,17,25,36] included both volunteers and mannequin heads but only the results drawn from the volunteers were considered within the scope of this review. In terms of the image acquisition technology, the 3D face-scanning systems used in all studies were classified into three major categories, namely, stereophotogrammetry, laser scanning, and structured light (Supplementary Materials Table S2). Eighteen studies evaluated multiple scanning devices. While most stationary scanning systems used belonged to the stereophotogrammetry category, the portable scanning devices conversely fell more equally into all three categories, i.e., stereophotogrammetry in ten studies, laser scanning in eight studies, and structured light in eight studies.

The reference images were made with stereophotogrammetry (*n* = 10 studies), direct anthropometry (*n* = 6 studies), computed tomography (*n* = 3 studies), laser scanning (*n* = 1 study), and structured light (*n* = 1 study). The number of facial landmark points compared ranged from 6 to 19 landmarks, with 5–136 linear distances. In one study [38], a test specimen attached to volunteers’ faces was used as the reference object to evaluate the accuracy of the facial scanning methods.

### 3.4. Meta-Analysis

**Figure 3:**
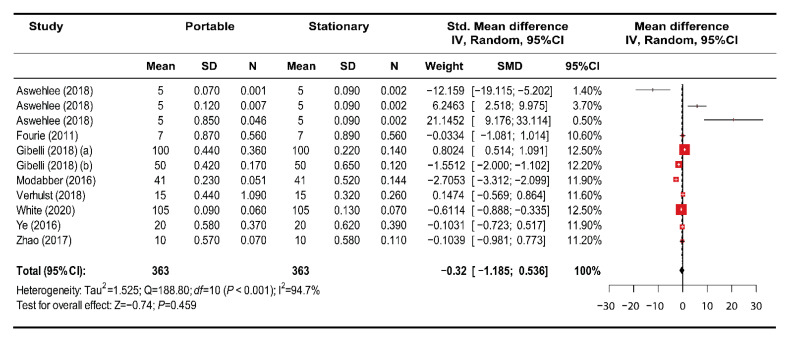
Meta-analysis results of the comparison between the use of portable and stationary face-scanning systems for generating digital face models.

**Figure 4:**
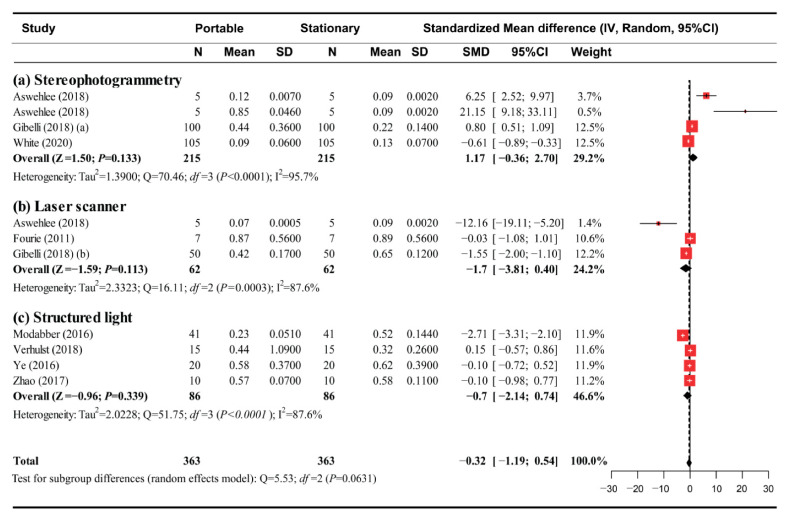
Meta-analysis results of the subgroup analysis. (**a**) Stereophotogrammetry; (**b**) laser scanner; (**c**) structured light.

Among the 21 studies, nine studies with a low risk of bias were included in the meta-analysis. The articles investigated both stationary and portable face-scanning systems and provided sufficient data for the pool-weighted estimation of Cohen’s d. In a global analysis, a random-effects model was selected to analyze the outcomes of the studies given the heterogeneity among them (Figure 3). Overall, the accuracy of the digital face models generated by the portable scanners was not significantly different from that of the stationary face-scanning systems (SMD (95% confidence interval) = −0.325 mm (−1.186 to 0.536); z = −0.74; *p* = 0.459). During the subgroup analysis for different image-capture types of portable face scanners, there was no significant difference noted in the estimated SMD between subgroups (*p* = 0.063). Within the subgroups, no statistical difference was found between the portable and stationary face-scanning systems (Figure 4), i.e., the stereophotogrammetry group (SMD (95% CI): 1.174 (−0.355 to 2.703); z = 1.50; *p* = 0.133), the laser scanning group (SMD (95% CI): −1.702 (−3.805 to 0.402); z = −1.59; *p* = 0.113), and the structured light group (SMD (95% CI): −0.702 (−2.141 to 0.736); z = −0.96; *p* = 0.339).

**Figure 5:**
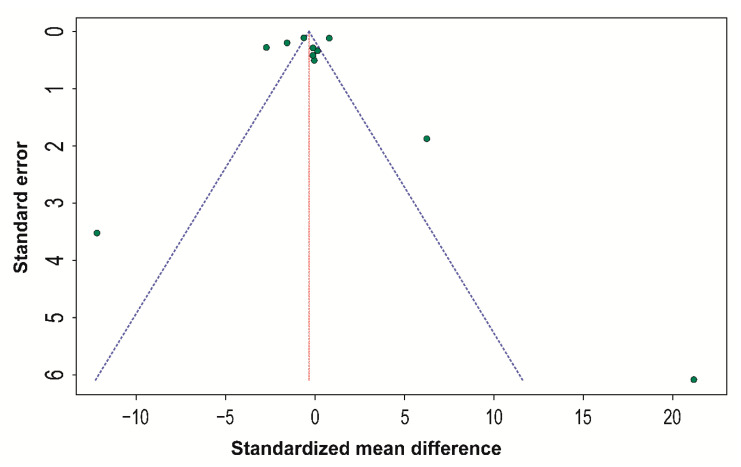
Funnel plot for the publication bias of all studies included in the meta-analysis.

Regarding the publication bias, funnel plotting and Egger’s regression test showed a low risk of publication bias for both the global and subgroup analyses (*p* = 0.958 for global analysis and *p* = 0.419, *p* = 0.781, and *p* = 0.491 for the analysis of the stereophotogrammetry, laser scanning, and structured light subgroups, respectively) (Figure 5).

## 4. Discussion

The meta-analysis revealed that the accuracy of the digital face models generated by portable face-scanning systems was comparable to that of those acquired using stationary systems. Within the comparison of the portable systems, no statistically significant difference was found regarding the accuracy of the facial models generated using the various scanning methods. The mean discrepancy values of the face models obtained by portable facial scanners were below 1.0 mm, which is considered acceptable for clinical use [1,40,44]. Portable face scanners relying on laser or structured light technology showed more diversity than those that rely on stereophotogrammetry technology [11,12,13,18,25,28,29,42,43].

Stereophotogrammetry technology captures surface images of the faces based on the multiple photoshoots taken by single-lens reflex cameras [24,27]. The software combines information about the camera position and camera-to-subject distance and calculates the 3D coordinates of each pair of 2D pixel points visible in different camera views by using specific algorithms to compile the 3D shape data [7]. As a result, the face models are generated with facial geometry represented as a dense cloud of points [45]. The major advantage of the stereophotogrammetry method is its ability to generate highly realistic, colored face models that resolve details of facial nature patterns, such as skin textures, pores, freckles, scars, and wrinkles, to represent the face [12]. However, the accuracy of the reconstructed images largely depends upon the integrity of the pixels, the resolution of the cameras, and the photo-taking conditions [11]. During image capture, strong, direct, ambient light may provoke a glare effect that dismisses or muddies the details of surface structures [46]. Therefore, light conditions must be carefully controlled by applying standardized flash units to eliminate interference from ambient spectral light. The system also requires a critical camera setting for shutter speed, brightness level, and aperture to control the quality of image exposure [7].

Laser and structured-light technology share similar working principles in this context because the two techniques both use an active image capture strategy that requires one to conduct only a single scan to obtain details of the face structures [47,48]. Laser scanners work by projecting a laser point or line onto the surface and capturing the light reflections with sensors [35]. Similarly, structured-light scanners project a pattern of light on the subject and use sensors or cameras to recognize the deformation of the pattern on the subject [15]. With a known distance from the light sources, a software algorithm is used to calculate the reflection angle of the light beam or the distance at each point in the light pattern to build up the triangulated geometry information of the structures [49]. The advantage of the active approach is the use of light projection to enhance the accuracy of facial surface mapping [50]. Moreover, typically, no additional light is needed during image acquisition because the lighting conditions are fully controlled by the systems, restricting the ambient lighting influence [7]. However, because the technique relies on capturing the light reflection with sensors, the presence of light-reflective or transparent surfaces could be quite problematic for achieving success using this technology [50].

Overall, motion artifacts were considered the main source of error in the results of portable face-scanning systems [13,15,25], cautioning that the influence of involuntary facial movements has a greater impact on portable face-scan devices than stationary ones. Prolonged scanning time and unstable movements of the scanners may magnify the motion artifacts caused by involuntary facial movements [15]. Therefore, the use of scanners that conduct a single and quick scan is recommended, especially when the face scans are performed on children or people with special needs who show difficulty in staying immobile for a prolonged period of time [15,23]. Caution should also be taken when scanning the facial regions that are more susceptible to involuntary movements, such as the eyes and mouth [13].

Within the included studies, the measurements of inter-landmark linear distance and surface-to-surface deviation were the most adopted methods for evaluating the accuracy of the digital facial models. For the inter-landmark linear distance, a clear landmark definition and the marking of prior landmarks are suggested to improve the accuracy of the measurements [34,37,40,41]. Landmarks based on well-defined border regions are preferred over landmarks that are based on gently curving slopes [16,35]. In stereophotogrammetry systems, facial surface texture with realistic color can be obtained, thus, facial landmarks can be simply identified by using ink markers [51]. For laser and structured-light scanning systems where high-resolution color information cannot be captured, different strategies were adopted to enhance the accuracy of landmark identification [40]. The use of protruded markers, such as opaque glass spheres [34] and adhesive stickers [29,40], was suggested as fiducial markers to eliminate landmark identification errors. Separately, automatic landmark localization obtained by curvature analysis was introduced to eliminate the subjective errors made by the manual landmark marking process [37]. For facial volume analysis and surface-to-surface distance, the accuracy in different facial regions was found to be inconsistent for 3D facial models, particularly for the face with deformities [1,12,18,52,53]. A smaller discrepancy was found in the frontal parts of the face, whereas a greater discrepancy was found in the lateral parts of the face, sides of the nose, and around the facial deformities [12,18].

The present review was limited to considering only 10 years of publications. This period was selected based on the assumption that papers published more than a decade ago may not reflect the nature of current scanning systems considering the rapid technological development that has occurred over time. Another limitation of this review was that the included studies did not directly compare the effects of the use of face-scanning diagnosis tools in terms of clinical treatment outcomes. However, the findings may assist the clinicians in making decisions on the use of face-scanning systems in the diagnosis phase. A wide variety was found among the portal face-scanning systems in the image acquisition and 3D reconstruction methods. Accordingly, each system requires a unique scanning protocol with different advantages and limitations that may affect the reliability and applicability in different ways. Further original studies and reviews need to extend the understanding of the accuracy of face scanner systems in more clinical conditions.

## 5. Conclusions

Within the limitations of this systematic review and meta-analysis, no significant difference was found between stationary and portable face-scanning systems with respect to the accuracy of the resultant digital face models. All investigated portable face scanners fell within a suitable range for clinical use. Within the comparison of scanning methods, stereophotogrammetry, laser, and structured-light systems showed similar levels of accuracy in generating a digital face model. The literature review revealed that scanning device settings, control of involuntary facial movements, landmark and facial region identifications, and scanning protocols are major factors that can affect the accuracy of face-scanning systems.

## Figures and Tables

**Table 1:** Inclusion and exclusion criteria for this study.

Study Characteristics	Inclusion Criteria	Exclusion Criteria
Study design	Systematic reviews, randomized and nonrandomized controlled trials, cohort studies, case-control studies, cross-sectional studies	Conference papers, case reports, letters, epidemiologic studies, narrative reviews, author ideas, editorials, opinion articles
Participants	Human faces of any age, inanimate objects reproducing the true human face	Studies that used only mannequin heads or phantoms
Interventions	Stationary and porTable 3D optical scanners (e.g., stereophotogrammetry, laser, structured light)	Studies that used only 2D images, studies with no use of porTable 3D face scanners
Measurements	Linear distances, surface-to-surface distance, surface areas, volumes	Studies without numeric measurements
Outcomes measures	Reliability (accuracy) of facial measurements obtained by porTable 3D face scanners versus stationary 3D face-scanning devices	Studies where the accuracy or precision of the facial scanning could not be determined

**Table 2:** Characteristics of the included studies.

Study	Scanner	No. Face	No. Landmark	Measurements	Conclusions
Aswehlee (2018)	Vivid 910, Danae 100SP, 3dMDface, Scanify	1 IC	3D point clouds	Surface deviation	Digitization of facial defect models using various noncontact 3D digitizers appears to be feasible and is most accurate with laser-beam, light-sectioning technology.
Bakirman (2017)	NA	2 (1M, 1F)	3D point clouds	Surface deviation	The outcome of the presented study showed that low-cost structured light scanners have great potential for 3D object modeling, including the human face.
Camison (2018)	3dMDface, Vectra H1	26 (6M, 20F), 1 MH	17; 3D point clouds	136 linear distances, Surface deviation	The results indicated that 3D facial surface images acquired with the Vectra H1 system are sufficiently accurate for most clinical applications.
Elbashti (2019)	Vivid 910, iPhone	1 IC	3D point clouds	Surface deviation	The results showed that, within the limits of this study and in reference to standard computed tomography imaging, data acquisition with a smartphone for 3D modeling is not as accurate as commercially available laser scanning.
Fourie (2011)	Di3D, Vivid 900	7 cadaver heads (NA)	15	21 Linear distances	Measurements recorded by the three 3D systems appeared to be both sufficiently accurate and reliable enough for research and clinical use.
Germec-Cakan (2010)	ZScanner 700, 3dMDface	15 (6M, 9F)	15	11 Linear distances	Laser scanning is not sensitive enough to visualize the deeper indentations, such as nostrils. Stereophotogrammetry is promising for 3D facial measurements and would be even better when color identification between mucocutaneous junctions of the lip region is achieved.
Gibelli (2018) (a)	Vectra M3, Vectra H1	50 (16M, 34F), 1 MH	12; 3D point clouds	15 Linear distances/12 angles/volumes/surfaces	The portable face scanning device (VECTRA H1) proved reliable for assessing linear measurements, angles, and surface areas; conversely, the influence of involuntary facial movements on volumes and root mean square (RMS) errors was higher compared with the static device.
Gibelli (2018) (b)	Vectra M3, Sense	50 (10M, 40F), 1 MH	17; 3D point clouds	14 Linear distances/12 angles/volumes/surfaces	The low-cost laser scan device can be reliably applied to inanimate objects, but does not meet the standards for three-dimensional facial acquisition on living persons.
Kim (2018)	Vectra M3, Vectra H1	5 (NA)	29	25 linear distances	The 3D handheld camera showed high accuracy and reliability in comparison with traditional models, indicating that this system may provide a useful tool in craniofacial anthropometry.
Knoops (2017)	3dMDface, M4D scan, Structure Sensor	8 (4M, 4F)	4; 3D point clouds	Surface deviation	Clinical and technical requirements govern scanner choice, however, 3dMDface System and M4D Scan provide high-quality results. It is foreseeable that compact, handheld systems will become more popular in the near future.
Koban (2020)	Vectra XT, Artec Eva, Sense	30 (15M, 15F), 1 MH	3D point clouds	Surface deviation	The 3D surfaces captured by a professional surface scanner (Artec EVA) showed a similarly desirable accuracy for facial imaging as VECTRA XT reference images. This handheld device presents a suitable option for the objective documentation during rhinoplasty surgery. Sense 3D was unable to accurately capture complex facial surfaces and is therefore limited in its usefulness for intraoperative mobile three-dimensional surface imaging (3DSI).
Lippold (2014)	FastSCAN	15 (5M, 10F)	12	7 Linear distances	The three-dimensional laser-scanning method using a laser scanner (FastSCAN) allowed a reliable and accurate identification of anatomical landmarks of the face. The obtained distances between certain landmarks, such as the intercanthal distance, were largely consistent with those from manual measurements.
Liu (2019)	Vectra H1, Scanify	2 (M)	13	11 Linear distances	Scanify is a very low-cost device that could have clinical applications for facial imaging if imaging errors are corrected by a future software update or hardware revision.
Maues (2018)	Di3D, Microsoft Kinect	10 (5M, 5F)	10	7 Linear distances, Surface deviation	Kinect showed good precision and reasonable accuracy, and appears to be an interesting and promising resource for facial analysis.
Modabber (2016)	Artec EVA, FaceScan3D	41 (16M, 25F)	2 Lego brick	Surface deviation	Scanning with Artec EVA led to more accurate 3D models compared to scanning with FaceScan3D. The exactness achieved by both scanners was comparable to other scanners mentioned in literature.
Piedra-Cascón (2020)	Bellus Face Camera Pro	10 (2M, 8F)	6	5 Linear distances	The facial digitizing procedure evaluated produced clinically acceptable outcomes for virtual treatment planning. The inter-examiner reliability between the 2 operators was rated as excellent, suggesting that the type of facial landmark used in this study provides reproducible results among different examiners.
Savoldelli (2019)	Vectra H1	2 (1M, 1F)	11	23 Linear distances	This study shows that the VECTRA H1 provides an accurate linear assessment of clinical parameters and allows the accurate analysis of craniofacial morphology. Furthermore, this device costs less and requires less space than other multi-pod devices
Verhulst (2018)	3dMDface, Vectra XT, Artec Eva	15 (6M, 9F)	3D point clouds	Surface deviation	All three imaging devices showed high reproducibility and accuracy. Although the Artec EVA showed a significant lower reproducibility, the difference found was not clinically relevant. Therefore, using these different systems alongside each other in clinical and research settings is possible.
White (2020)	3dMDface, Vectra H1	35 (N/A), 1MH	19, 3D point clouds	Surface deviation	When the two camera systems were used separately to image human participants, this analysis found an upper bound of error potentially introduced by the use of the 3dMDface or VECTRA H1 camera systems, in conjunction with the MeshMonk registration toolbox, at 0.44 mm and 0.40 mm, respectively. For studies using both camera systems, this upper bound increased to 0.85 mm on average, and there were systematic differences in the representation of the eyelids, nostrils, and mouth by the two camera systems.
Ye (2016)	3D CaMega, 3dMDface	10 (5M, 5F)	16	21 Linear distances	When applied in scanning and measuring human faces, the structured light scanning system and stereophotogrammetry scanning system both demonstrated high accuracy, reliability, and reproducibility.
Zhao (2017)	Faro, 3dMDface, FaceScan3D	10 (NA)	3D point clouds	Surface deviation	The 3D accuracy of different facial partitions was inconsistent; the middle face had the best performance. Although the practical accuracy of two facial scanners was lower than their nominal accuracy, they all met the requirement for oral clinic use.

3D = three-dimensional; RCT = Randomized Controlled Trial; M = male; F = female; IC = impression cast; MH = mannequin head; NA = none available. Manufacturing information for all the products listed in this table are provided in the Supplementary Materials Table S2 of this study.

## Data Availability

Data is contained within the article.

## Supplementary Materials

The following are available online at https://www.mdpi.com/1660-4601/18/1/94/s1, Table S1: Individual search strategies; Table S2: Stationary and portable face-scanning commercial devices investigated in the included studies.
