# Supplementary material for: Accuracy of Portable Face-Scanning Devices for Obtaining Three-Dimensional Face Models: A Systematic Review and Meta-Analysis
Source: Int J Environ Res Public Health. 2020 Dec 25;18(1):94. doi: 10.3390/ijerph18010094 (PMC7795319; doi:10.3390/ijerph18010094)
Supplement: Supplementary file 1 [file ijerph-18-00094-s001.zip › Supplementary Materials Table 1.pdf]

Supplementary Materials Table 1. Formulated Boolean operators incorporated in each searching strategy.

Database	Boolean Operator	Result
Pubmed	(((((((("virtual patient") OR "virtual face") OR "virtual facial model") OR "digital face") OR "digital facial models") OR "three-dimensional face image") OR "facial appearance" [MeSH Terms]) OR "face" [MeSH Terms]) AND (((((((((((((("scanner" [MeSH Terms]) OR "facial scan") OR "digital face scan") OR "3D facial scan") OR "3D face impression") OR "indirect face capturing") OR "direct face capturing") OR "face digitalization") OR "3D face scanning") OR "computer-assisted face assessment") OR "structured light method") OR "moir fringe method") OR stereophotography) OR photogrammetry) OR "white-light scanner") OR "optical scanners") AND (((((((("dimensional measurement accuracy" [MeSH Terms]) OR accuracy) OR precision) OR trueness) OR feasibility) OR reliability) OR "3D comparison") OR "data accuracy" [MeSH Terms])	1050
Scopus	TITLE-ABS-KEY ("virtual patient" OR "virtual face" OR "virtual facial model" OR "digital face" OR "digital facial models" OR "three-dimensional face image" OR "facial appearance" OR "face") AND TITLE-ABS-KEY ("scanner" OR "facial scan" OR "digital face scan" OR "facial scan" OR "3D face impression" OR "indirect face capturing" OR "direct face capturing" OR "face digitalization" OR "3D face scanning" OR "computer-assisted face assessment" OR "structured light method" OR "moir fringe method" OR stereophotography OR photogrammetry OR "white-light scanner" OR "optical scanners") AND TITLE-ABS-KEY ("dimensional measurement accuracy" OR accuracy OR precision OR trueness OR feasibility OR reliability OR "3D comparison" OR "data accuracy")	847
Cochrane	"virtual patient" OR "virtual face" OR "virtual facial model" OR "digital face" OR "digital facial models" OR "three-dimensional face image" AND "scanner" OR "facial scan" OR "digital face scan" OR "facial scan" OR "3D face impression" OR "indirect face capturing" OR "face digitalization" OR "3D face scanning" OR "computer-assisted face assessment" OR "structured light method" OR "moir fringe method" OR stereophotography OR photogrammetry OR "white-light scanner" OR "optical scanners" AND "dimensional measurement accuracy" OR accuracy OR precision OR trueness OR feasibility OR reliability OR "3D comparison" OR "data accuracy"	458
Science Direct	("digital face scanning" OR "facial scan" OR "3D face scan" OR" optical face scanners") and ALL(accuracy OR trueness OR "dimensional measurement accuracy" OR precision)	257
Google Scholar	"digital face scanning" OR "3D facial scan" OR "optical face scanners" AND "accuracy" OR "trueness" OR "dimensional measurement accuracy" OR "precision"	194
**Total**		**2806**
