# Supplementary material for: Accuracy of Portable Face-Scanning Devices for Obtaining Three-Dimensional Face Models: A Systematic Review and Meta-Analysis
Source: Int J Environ Res Public Health. 2020 Dec 25;18(1):94. doi: 10.3390/ijerph18010094 (PMC7795319; doi:10.3390/ijerph18010094)
Supplement: Supplementary file 1 [file ijerph-18-00094-s001.zip › Supplementary Materials Table 2.pdf]

Supplementary Materials Table 2. Stationary and portable face-scanning commercial devices investigated in the included studies.

Image capture technology	Stationary*	Portable*	Study
Stereophotogrammetry (n=27)	3dMDface (3dMD LLC, Atlanta, USA)		Aswehlee (2018), Camison (2018), Germec-Cakan (2010), Knoops (2017), Verhulst (2018), White (2020), Ye (2016), Zhao (2017)
	FaceScan3D (3D Shape GmbH, Erlangen, Germany)		Modabber (2016), Zhao (2017)
	Di3D (Dimensional Imaging, Glasgow, UK)		Fourie (2011), Maues (2018)
		M4D Scan (Rodin4D, Pessac, France)	Knoops (2017)
		Scanify (Fuel 3D Technologies Limited)	Aswehlee (2018), Liu (2019)
	Vectra XT (Canfield Scientific Inc., Parsippany-Troy Hills, NJ, USA)		Koban (2020), Verhulst (2018)
	Vectra M3 (Canfield Scientific, Fairfield, NJ, USA)		Gibelli (2018) (a), Gibelli (2018) (b), Kim (2018)
	Danae 100SP (NEC Engineering)		Aswehlee (2018)
		Vectra H1 (Canfield Scientific, Parsippany, NJ, USA)	Camison (2018), Gibelli (2018) (a), Kim (2018), Liu (2019), Savoldelli (2019), White (2020)
Laser scanner (n=8)		Vivid 900 (Minolta Co., Ltd., Osaka, Japan)	Fourie (2011)
		Vivid 910 (Minolta Co., Ltd., Osaka, Japan)	Aswehlee (2018), Elbashti (2019)
		Sense (3D Systems, Rock Hill, SC, USA)	Gibelli (2018) (b), Koban (2020)
		Faro (Faro Edge LLP, Faro, Florida, USA)	Zhao (2017)
		ZScanner 700 (Z Corporation, Burlington, MA)	Germec-Cakan (2010)
		FastSCAN (Polhemus, Colchester, Vermont, USA)	Lippold (2014)
Structured light (n=8)		Structure Sensor (Occipital Inc., San Francisco, CA, USA)	Knoops (2017)
		Microsoft Kinect (Microsoft Corporation, Redmond, WA, USA)	Maues (2018)
		Artec Eva (Artec Group, Luxembourg, Luxembourg)	Koban (2020), Modabber (2016), Verhulst (2018)

		Face Camera Pro Bellus (Face Camera Pro Bellus; Bellus3D)<br>3D CaMega (BWHX Technology Company, Beijing, China)<br>iPhone (Apple Store, Cupertino, CA, USA)	Piedra-Cascón (2020)<br>Ye (2016)<br>Elbashti (2019)

*Manufacture information as provided in the included studies.
